# Author Correction: ATP hydrolysis by yeast Hsp104 determines protein aggregate dissolution and size in vivo

**DOI:** 10.1038/s41467-020-20394-8

**Published:** 2020-12-14

**Authors:** Udhayabhaskar Sathyanarayanan, Marina Musa, Peter Bou Dib, Nuno Raimundo, Ira Milosevic, Anita Krisko

**Affiliations:** 1 European Neuroscience Institute (ENI) – A Joint Initiative of the University Medical Center Göttingen and the Max-Planck-Society, Göttingen, Germany; 2 Mediterranean Institute for Life Sciences, Split, Croatia; 3 Institut für Zellbiochemie, Universitätsmedizin Göttingen, Göttingen, Germany; 4 Wellcome Centre for Human Genetics, Nuffield Department of Medicine, NIHR Oxford Biomedical Research Centre, University of Oxford, Oxford, OX3 7BN UK; 5 Department of Experimental Neurodegeneration, University Medical Center Göttingen, Göttingen, Germany

Correction to: *Nature Communications* 10.1038/s41467-020-19104-1, published online 16 October 2020.

The original version of this Article contained an error in the author affiliations.

Affiliation 1 incorrectly read ‘European Neuroscience Institute (ENI), Göttingen, Germany’ instead of the correct ‘European Neuroscience Institute (ENI)—A Joint Initiative of the University Medical Center Göttingen and the Max-Planck-Society, Göttingen, Germany.’

This has now been corrected in both the PDF and HTML versions of the Article.

