# Supplementary material for: ATP hydrolysis by yeast Hsp104 determines protein aggregate dissolution and size in vivo
Source: Nat Commun. 2020 Oct 16;11:5226. doi: 10.1038/s41467-020-19104-1 (PMC7568574; doi:10.1038/s41467-020-19104-1)
Supplement: Supplementary file 1 — Supplementary information [file 41467_2020_19104_MOESM1_ESM.pdf]

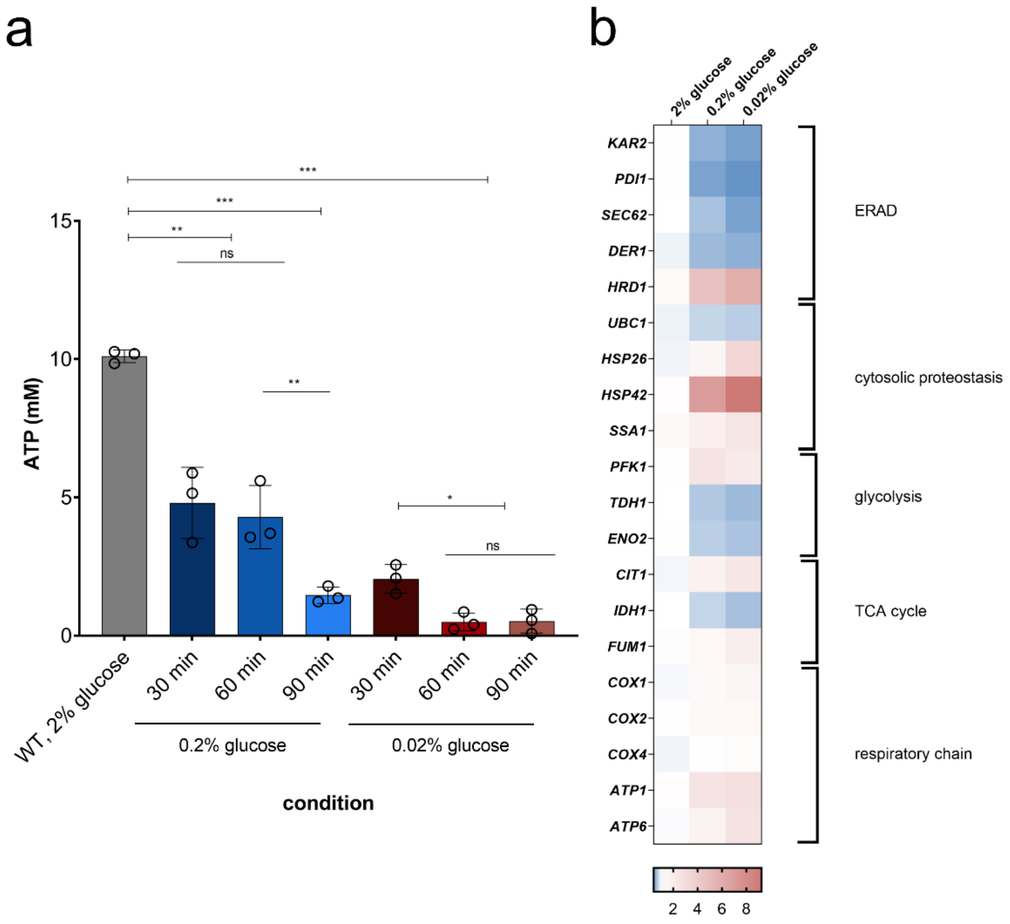

Supplementary Figure 1. Glucose starvation triggers ATP decline and the activation of heat shock response. (a) Glucose starvation in 0.2% glucose (blue) and 0.02% glucose (red) causes a decrease in cellular ATP levels compared to control (grey). Cells were starved for 90 minutes in mid-exponential phase and aliquots were taken at 30, 60 and 90 minutes. Bar heights are mean ± SD from 3 independent cultures, each performed in triplicate. The mean values of three technical replicates for each biological replicate are given as individual data points. *** $p<0.001$; ** $p<0.01$; * $p<0.05$ (One-way ANOVA plus Tukey post hoc). **(b)** qPCR measurement of differential gene expression reveals that glucose starvation response is accompanied by heat shock response activation, as well as the shift to respiratory metabolism. Cells were starved for 90 minutes in mid-exponential phase. Color of the squares on the heat map corresponds to the mean value of the log fold change from three biological and three technical replicates. Standard deviation and p values for the comparison to the control are available in the Source Data file. UBC6 was used for normalization.

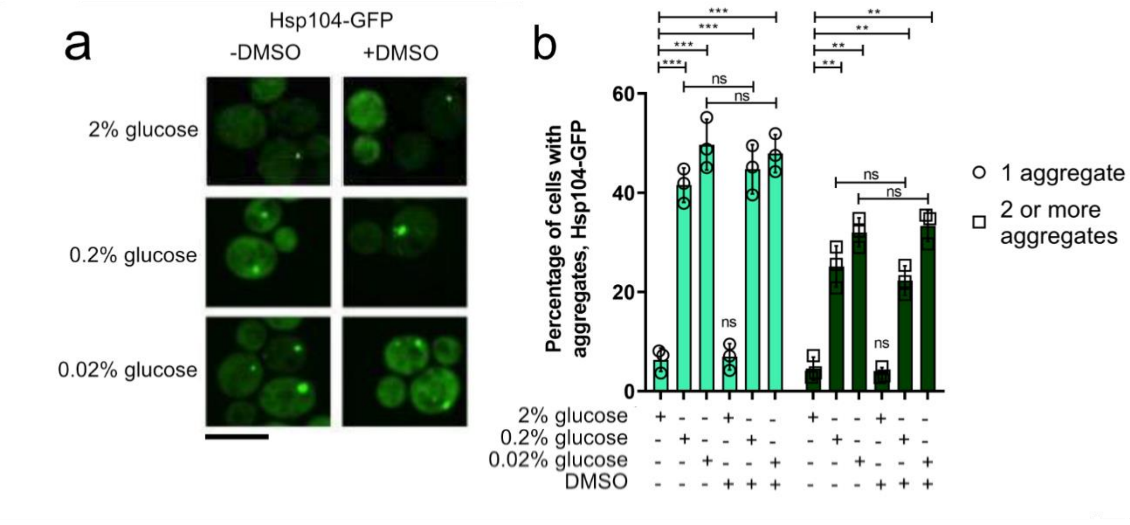

Supplementary Figure 2. Addition of DMSO does not influence protein Hsp104-GFP tagged aggregate formation. (a) Representative images of Hsp104-GFP containing aggregates in the presence of 5% DMSO (final). DMSO was added into the culture at the beginning of the starvation period. The images are representative of three technical replicates for each of the three biological replicates. Black bar represents 8 µm. **(b)** Percentage of cells with one (light green), and two or more aggregates (dark green) is not changed between untreated control (assessed in the same experiment) and after addition of DMSO, both in control, as well as in 0.2% and 0.02% glucose. N=550 cells were screened for aggregates starting from three independent exponential yeast cultures for each condition. Data are mean ± SD from at least 3 independent cultures, each performed in triplicate, and the means of technical replicates are represented as individual data points. *** $p<0.001$; ** $p<0.01$; * $p<0.05$ (One-way ANOVA plus Tukey post hoc).

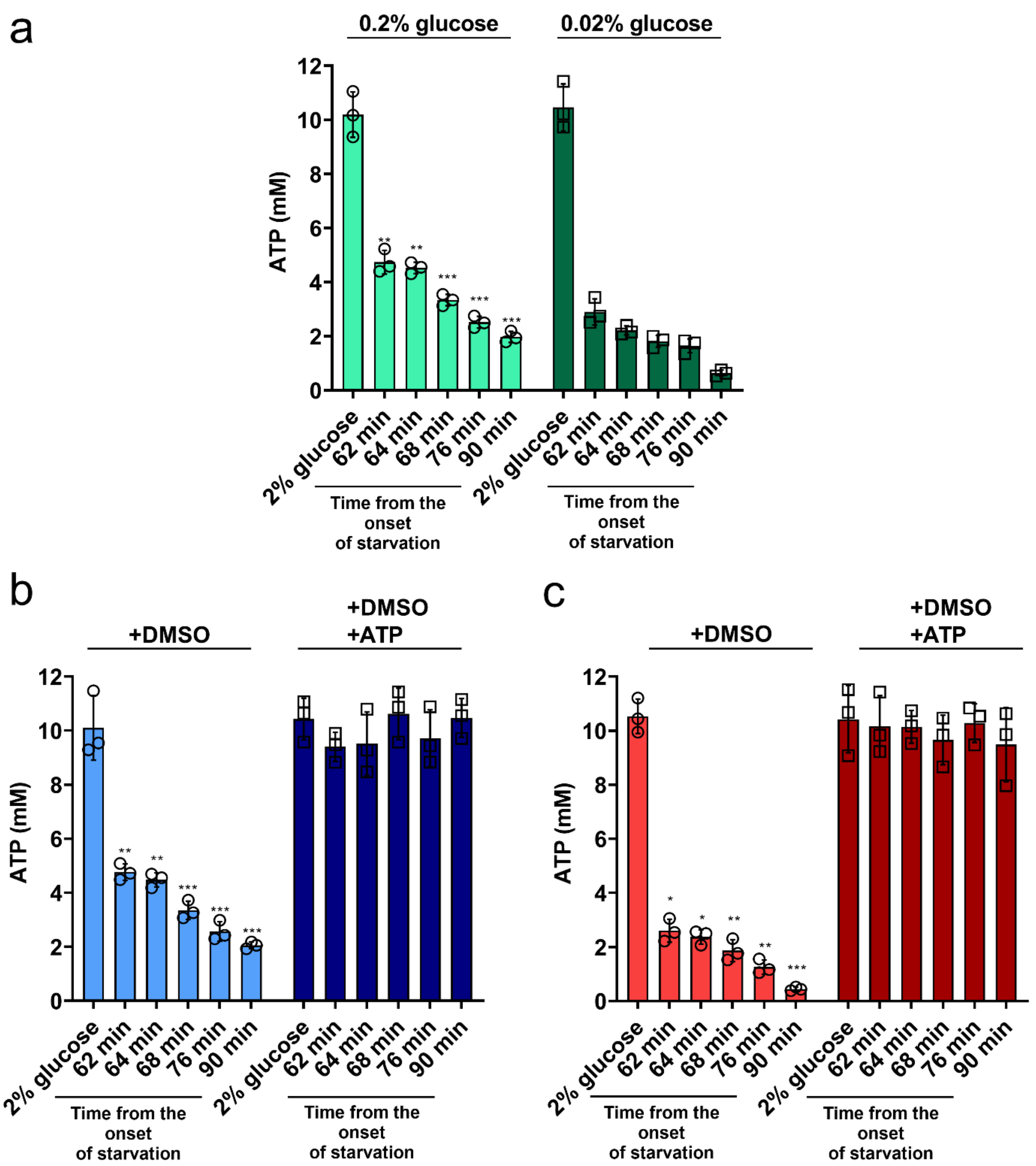

Supplementary Figure 3. **The presence of DMSO allows the entry of ATP into yeast cells. (a)** ATP levels decline during the starvation in 0.2% (light green, circles) and 0.02% glucose (dark green, squares). The cells were starved for 90 minutes in mid-exponential phase and aliquots were taken from the culture at indicated times to measure the ATP level. Cellular ATP levels in the presence of DMSO in **(b)** 0.2% glucose, and **(c)** 0.02% glucose starvation in the absence of external ATP (circles) and its presence (squares). DMSO and ATP were added into the culture simultaneously 60 minutes after the beginning of the 90-minute starvation period. Bar heights are mean ± SD from at least 3 independent cultures, each performed in triplicate, and the means of technical replicates are represented as individual data points. *** p<0.001; ** p<0.01; * p<0.05 (One-way ANOVA plus Tukey post hoc).

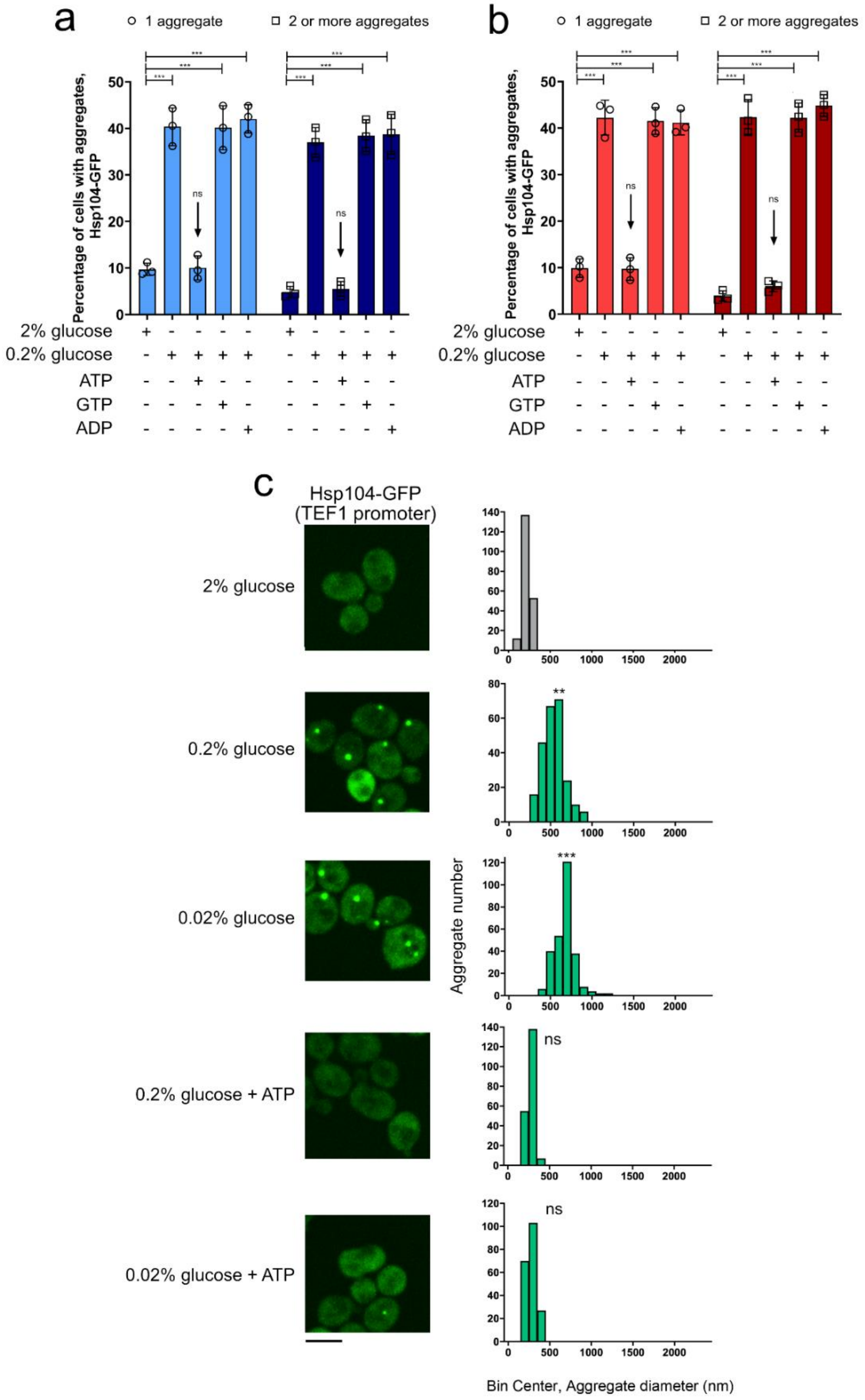

Supplementary Figure 4. Percentage of cells bearing aggregates increases almost six-fold during glucose starvation observed using Hsp104-GFP under the control of TEF1 promoter. This is observed in **(a)** 0.2% (blue) and **(b)** 0.02% (red) glucose medium. Treatment with ATP, but not GTP or ADP (all in

the presence of DMSO), causes the fraction of cells bearing aggregates to return to levels comparable to control. N=550 cells were screened for aggregates starting from three independent exponential yeast cultures for each condition. Data are mean ± SD from at least 3 independent cultures, each performed in triplicate, and the means of technical replicates are represented as individual data points. *** $p<0.001$; ** $p<0.01$; * $p<0.05$ (One-way ANOVA plus Tukey post hoc). **(c)** Mean aggregate diameter shifts to larger sizes during starvation in 0.2% and 0.02% glucose. Addition of ATP (in the presence of DMSO) results in aggregates reverting to the sizes observed in control conditions. Data represent binned values of individual aggregate diameters for N=202, 240, 275, 200, and 200 aggregates from three independent exponential yeast cultures for the conditions of: 2% glucose, 0.2% glucose, 0.02% glucose, 0.2% glucose+ATP, and 0.02% glucose + ATP, respectively. Kolmogorov-Smirnov test was used to compare the statistical significance of the observed differences between each studied condition with the control (2% glucose) (*** $p<0.001$; ** $p<0.01$; * $p<0.05$). Black bar represents 8 µm.

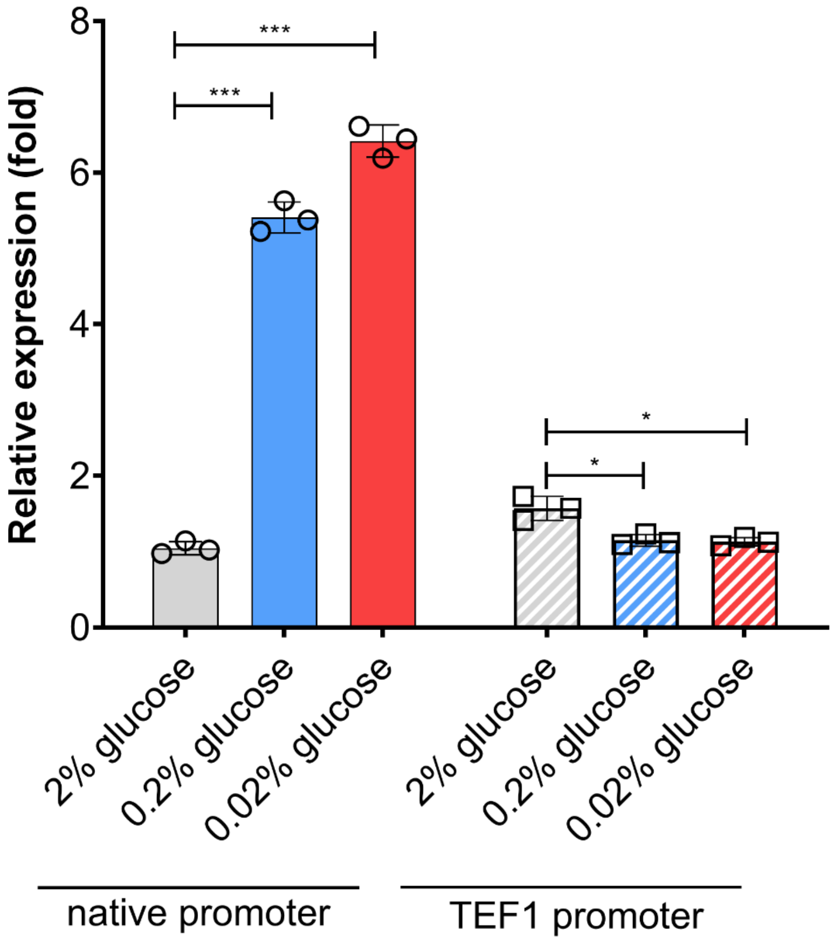

Supplementary Figure 5. qPCR measurement of Hsp104-GFP differential expression under the control of two different promoters. When under the control of the native promoter (circles), Hsp104 undergoes an increase in its expression level in starvation conditions, which is absent when Hsp104 is controlled by the TEF1 promoter (squares). Bar height represents mean ± SD from 3 separate cultures, each performed in triplicate. The mean values of three technical replicates for each biological replicate are given

as individual data points. UBC 6 was used for normalization. *** p<0.001; ** p<0.01; * p<0.05 (One-way ANOVA plus Tukey post hoc).

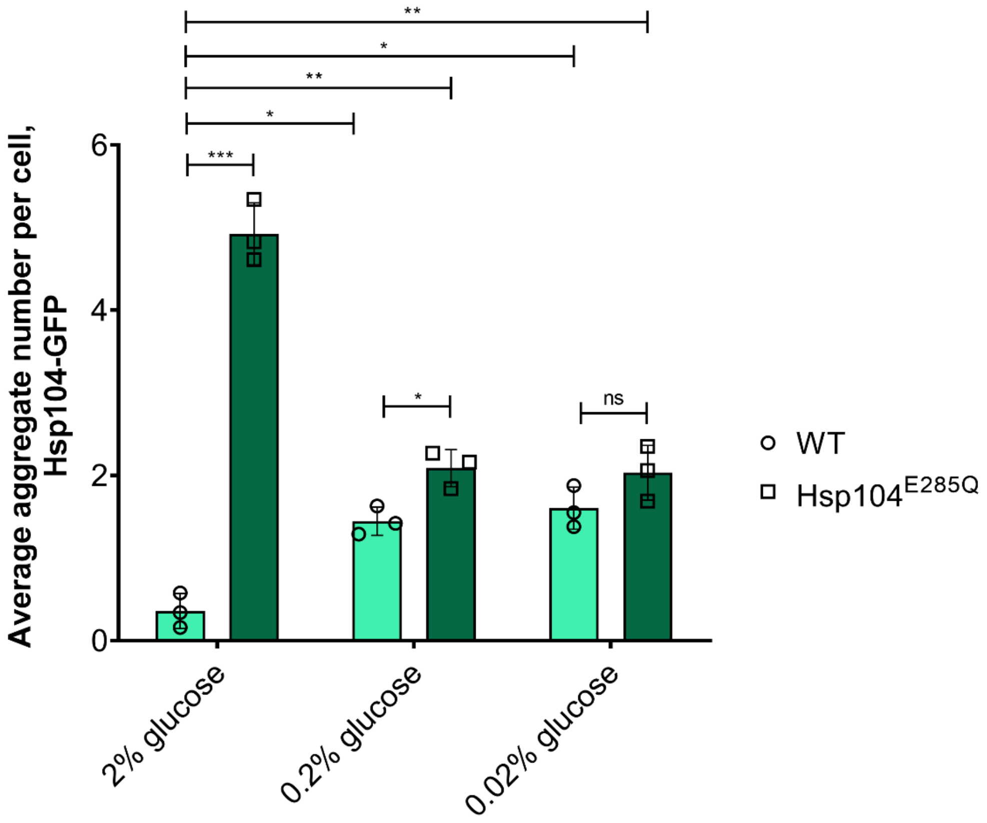

Supplementary Figure 6. Average aggregate per-cell number changes as a function of glucose availability. Average number of aggregates per cell increases during glucose starvation in the wild type strain (circles, light green bars), however, decreases in the strain expressing Hsp104^{E285Q} mutant (squares, dark green bars). N=550 cells were screened for aggregates starting from three independent exponential yeast cultures for each condition. Data are mean ± SD from at least 3 independent cultures, each performed in triplicate, and the means of technical replicates are represented as individual data points. *** p<0.001; ** p<0.01; * p<0.05 (One-way ANOVA plus Tukey post hoc).

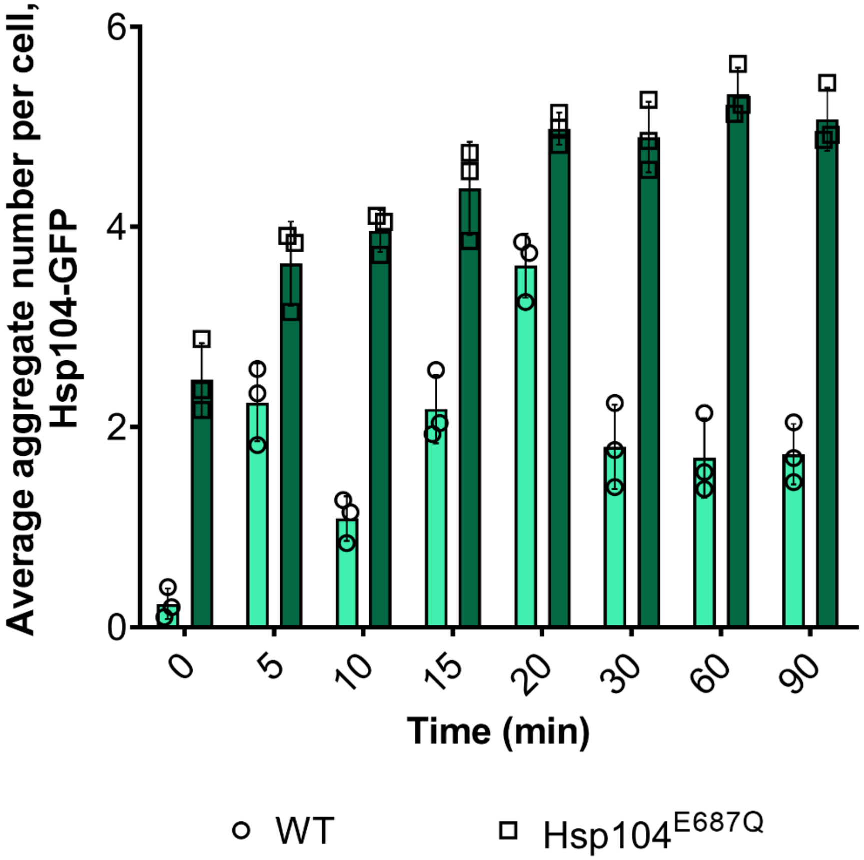

Supplementary Figure 7. Average aggregate per-cell number changes during glucose starvation. Average number of aggregates per cell varies during the time course of glucose starvation in the wild type strain (circles, light green bars), likely reflecting aggregate growth and fusion events. However, in the Hsp104^{E687Q} mutant (squares, dark green bars), this number steadily increases, likely due to inability to perform aggregate fusion. N=550 cells were screened for aggregates starting from three independent exponential yeast cultures for each condition. Data are mean ± SD from at least 3 independent cultures, each performed in triplicate, and the means of technical replicates are represented as individual data points. *** p<0.001; ** p<0.01; * p<0.05 (One-way ANOVA plus Tukey post hoc).

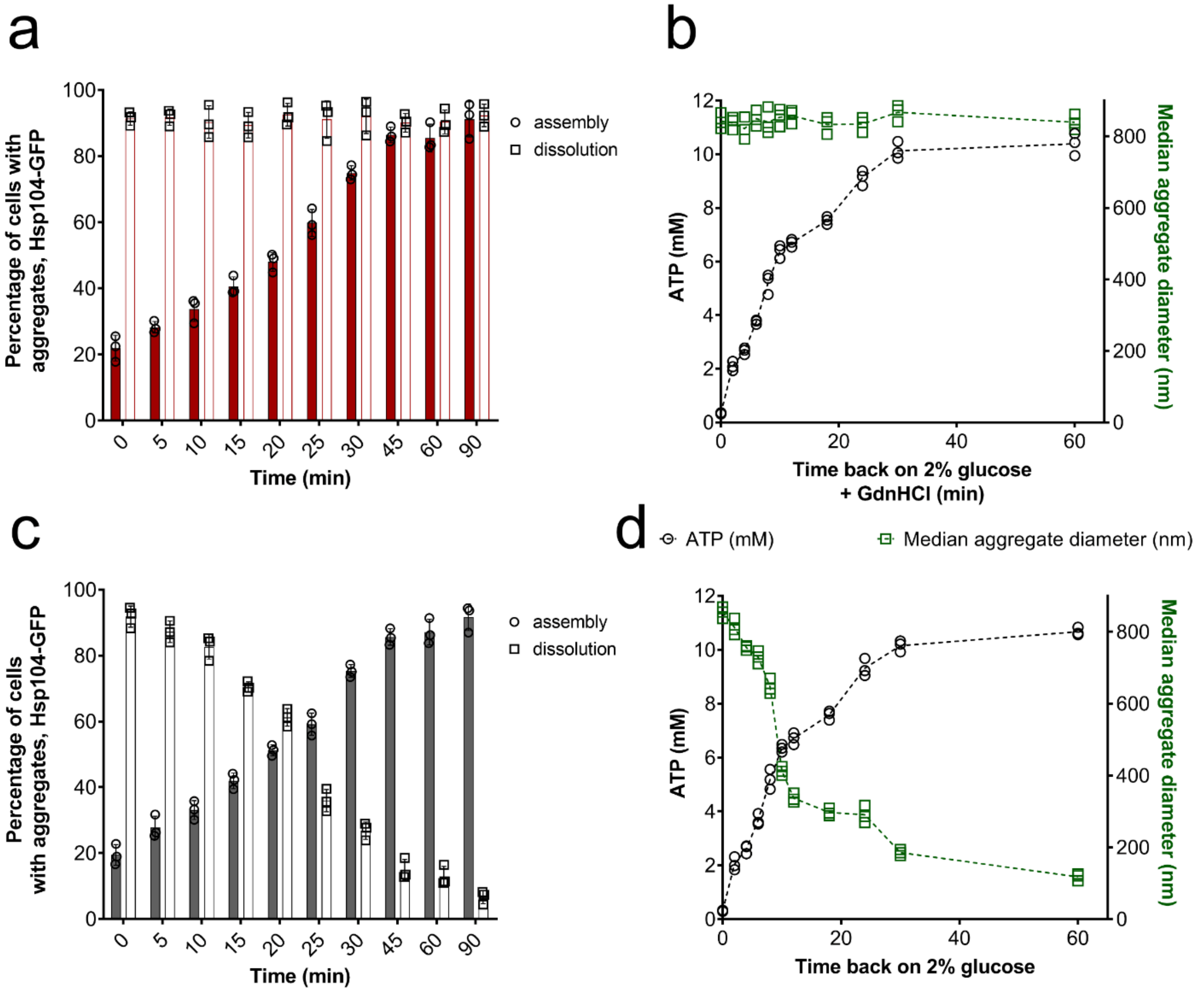

Supplementary Figure 8. Guanidinium hydrochloride (GdnHCl) inhibits the post-starvation dissolution of protein aggregates. (a) Percentage of cells displaying at least one Hsp104-GFP tagged aggregate increases during 0.02% glucose starvation (red bars, circles), but does not decrease back to control level during recovery in 2% glucose in the presence of GdnHCl (white bars, squares). GdnHCl was added into the culture simultaneously with glucose at the end of a 90-minute starvation period. N=550 cells were screened for aggregates starting from three independent exponential yeast cultures for each condition. Bar heights are mean ± SD from 3 independent cultures, each performed in triplicate. The mean of three technical replicates for each biological replicate is displayed as single data points. *** $p<0.001$; ** $p<0.01$; * $p<0.05$ (One-way ANOVA plus Tukey post hoc). **(b)** ATP levels (black circles) are restored during recovery in 2% glucose in the presence of GdnHCl, but the median aggregate diameter (green squares) fails to decrease to control levels. GdnHCl was added into the culture simultaneously with glucose at the end of a 90-minute starvation period. Individual data points are mean of three technical replicates of each of the biological replicate. **(c)** Percentage of cells displaying at least one Hsp104-GFP tagged aggregate (grey bars, circles) increases during 0.02% glucose starvation, and decreases back to control level during recovery in 2% glucose (white bars, squares). N=550 cells were screened for aggregates starting from three independent exponential yeast cultures for each condition. Bar heights are mean ± SD from 3 independent cultures, each performed in triplicate. The mean of three technical replicates for each biological replicate

is displayed as single data points. *** p<0.001; ** p<0.01; * p<0.05 (ANOVA plus Tukey post hoc). **(d)** ATP levels (black circles) are restored during recovery in 2% glucose, and the median aggregate diameter (green squares) decrease to control levels. Individual data points are mean of three technical replicates of each of the biological replicate.

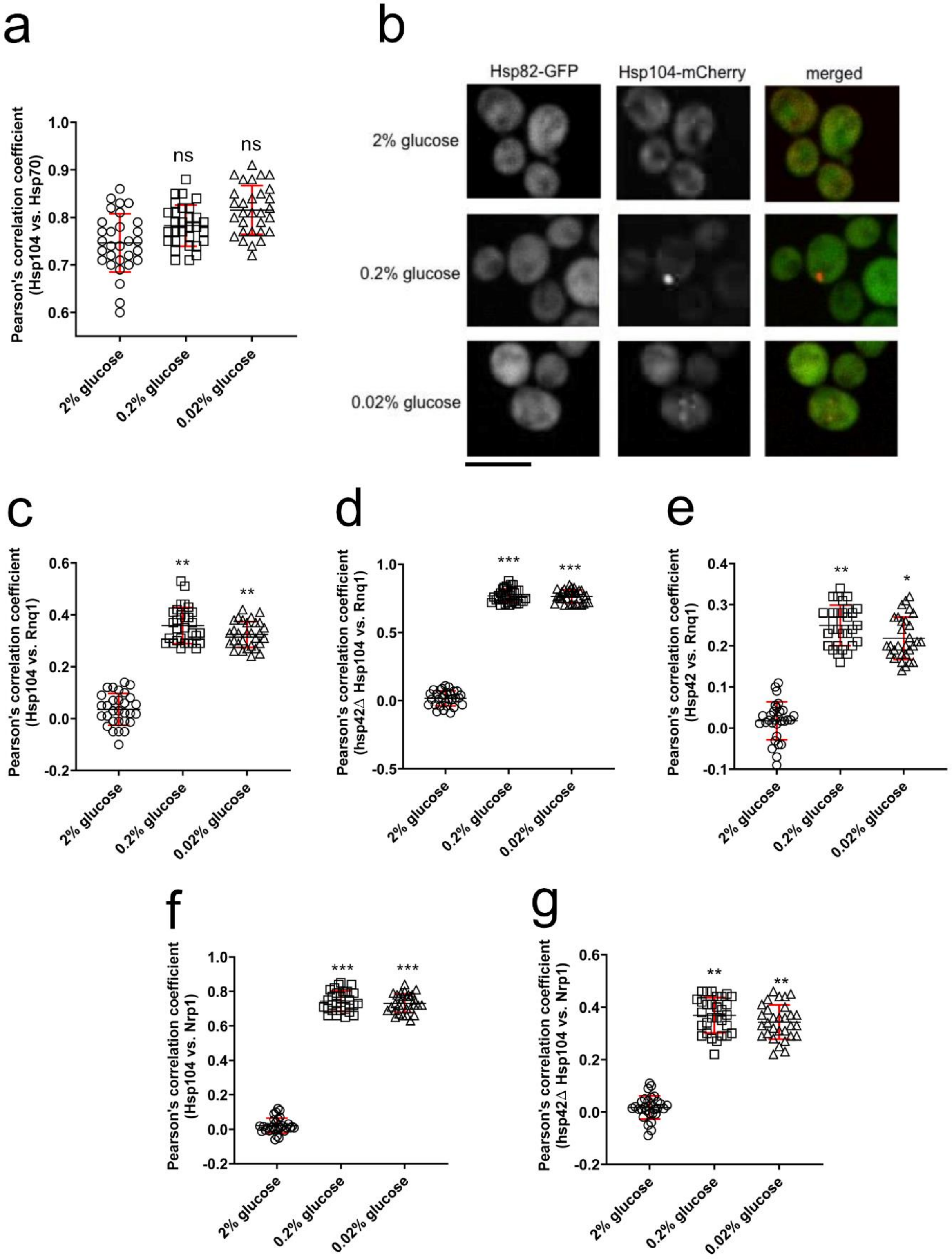

Supplementary Figure 9. Colocalization analyses reveal identity of protein aggregates formed during starvation. (a) Quantification of the colocalization between Hsp104 and Hsp70 using the Pearson's colocalization coefficient. **(b)** Hsp82-GFP does not form distinct puncta during glucose starvation. Hsp104-mCherry containing aggregates form with typical abundance. N=1200 cells were screened for aggregates starting from three independent exponential yeast cultures for each condition. Black bar represents 8 µm. Quantification of the colocalization between **(c)** Hsp104 and Rnq1 in the WT background; **(d)** Hsp104 and Rnq1 in the *hsp42Δ* background; **(e)** Hsp42 and Rnq1 in the WT background; **(f)** Hsp104 and Nrp1 in the WT background, and **(g)** Hsp104 and Nrp1 in the *hsp42Δ* background, using the Pearson's colocalization coefficient. Individual data points are displayed for each of the 30 images analyzed. Each image contained at least 50 cells. In all colocalization analyses, the center line represents the median, and the error bar (red) represents the standard deviation. The data is pooled from three different experiments. *** $p<0.001$; ** $p<0.01$; * $p<0.05$ (One-way ANOVA plus Tukey post hoc).

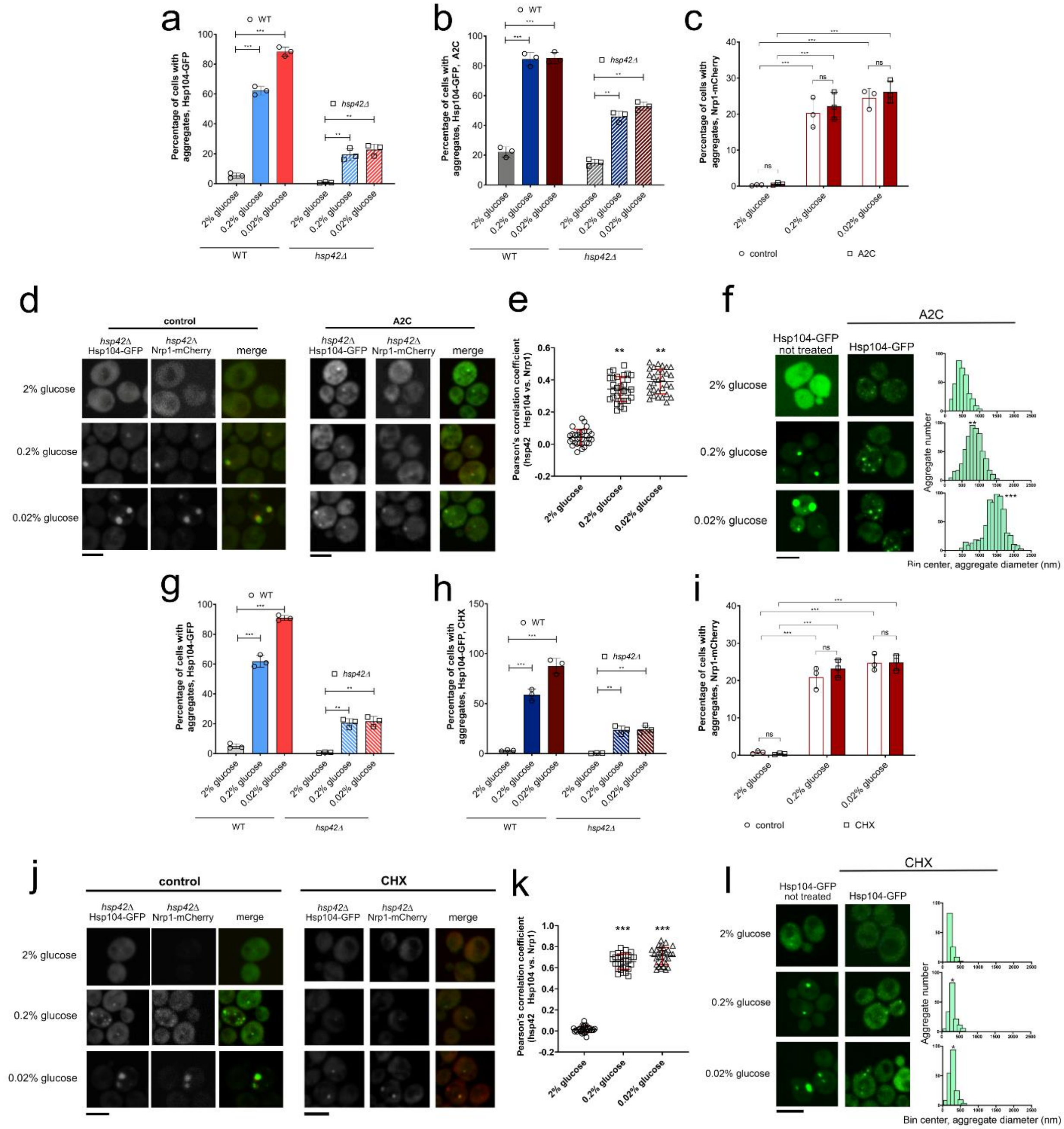

Supplementary Figure 10. Misfolded proteins and nascent polypeptides are secluded into aggregates during starvation.

(a) In the absence of A2C, ~60% and ~80% of WT cells display at least 1 aggregate in 0.2% and 0.02% glucose, respectively, as well as ~20% of *hsp42Δ* cells. **(b)** Exposure to A2C results in more than 80% of cells displaying at least 1 aggregate during starvation in the WT background, and ~50% in the *hsp42Δ* background. **(c)** Percentage of cells with Nrp1-mCherry foci in the *hsp42Δ* background is not affected by A2C. In **(a)** to **(c)**, bar heights are mean ± SD from at least 3 independent cultures, each performed in triplicate. Individual data points are mean values of three technical replicates for each biological replicate.

N=500 cells we screened for aggregates starting from three independent exponential yeast cultures for each condition. p<0.001; ** p<0.01; * p<0.05 (One-way ANOVA plus Tukey post hoc). **(d)** Representative images, and **(e)** colocalization analysis of Nrp1 and Hsp104 in the *hsp42Δ* using Pearson's colocalization coefficient. Individual data points are displayed for each of the 30 images analyzed. Each image contained at least 50 cells. In all colocalization analyses, the center line represents the median, and the error bar (red) represents the standard deviation. The data is pooled from three different experiments. Black bar represents 8 µm. **(f)** The median aggregate size increases in the presence of A2C from around 450 nm in the wild type control, to 900 nm in 0.2% glucose and 1500 nm in 0.02% glucose. Data represent binned values of individual aggregate diameters for N=664, 589, and 362 aggregates from three independent exponential yeast cultures for each condition: 2%, 0.2% and 0.02% glucose, respectively. Kolmogorov-Smirnov test was used to compare the statistical significance of the observed differences between each studied condition and the control (2% glucose) (*** p<0.001; ** p<0.01; * p<0.05). **(g)** In the absence, and **(h)** presence of CHX, ~60% and ~80% of WT cells display at least 1 aggregate in 0.2% and 0.02% glucose, respectively, as well as ~20% of *hsp42Δ* cells. **(i)** Percentage of cells with Nrp1-mCherry foci in the *hsp42Δ* background is not affected by CHX. In **(g)** to **(i)**, bar heights are mean ± SD from at least 3 independent cultures, each performed in triplicate. Individual data points are mean values of three technical replicates for each biological replicate. N=500 cells we screened for aggregates starting from three independent exponential yeast cultures for each condition. p<0.001; ** p<0.01; * p<0.05 (One-way ANOVA plus Tukey post hoc). **(j)** Representative images, and **(k)** colocalization analysis of Nrp1 and Hsp104 in the *hsp42Δ* using Pearson's colocalization coefficient. Individual data points are displayed for each of the 30 images analyzed. Each image contained at least 50 cells. In all colocalization analyses, the center line represents the median, and the error bar (red) represents the standard deviation. The data is pooled from three different experiments. Black bar represents 8 µm. **(l)** Median diameter of the aggregates formed during CHX treatment is ~300 nm in 0.2% and 0.02% glucose. Data represent binned values of individual aggregate diameters for N=121, 162, and 197 aggregates from three independent exponential yeast cultures for each condition: 2%, 0.2% and 0.02% glucose, respectively. Kolmogorov-Smirnov test was used to compare the statistical significance of the observed differences between each studied condition and the control (2% glucose) ***

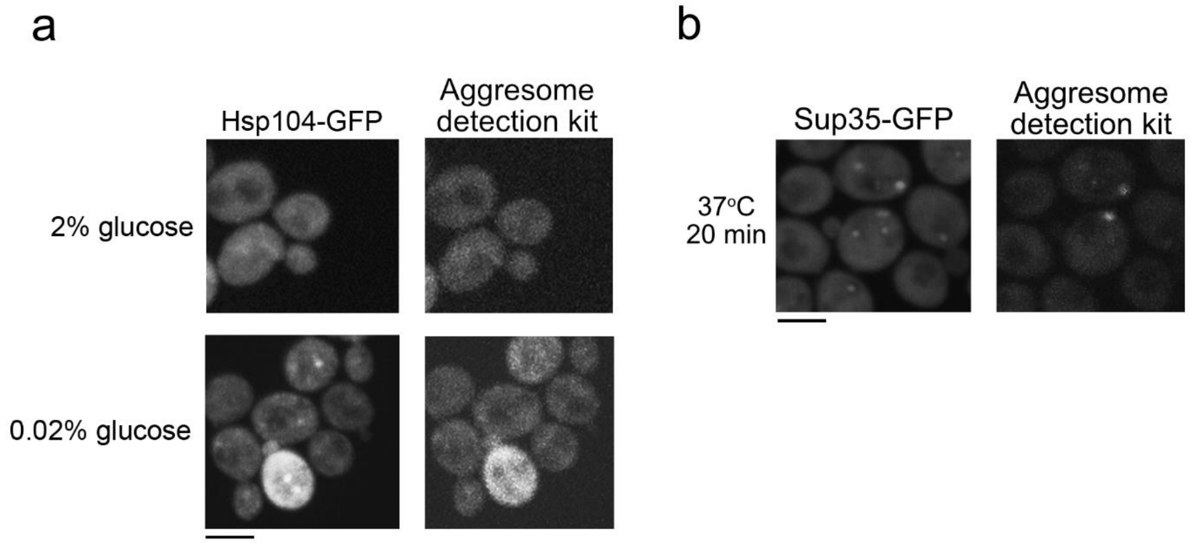

Supplementary Figure 11. (a) None of the Hsp104-GFP positive aggregates formed in 0.02% glucose stain with Aggresome detection kit (Abcam), suggesting the absence of amyloid structures. **(b)** As a positive control, a fraction of Sup35-GFP aggregates, formed during 37ºC heat shock for 20 minutes, were stained using the same method. Representative images for are presented. The images are representative of three technical replicates for each of the three biological replicates (~500 analyzed cells). Black bar represents 8 µm.

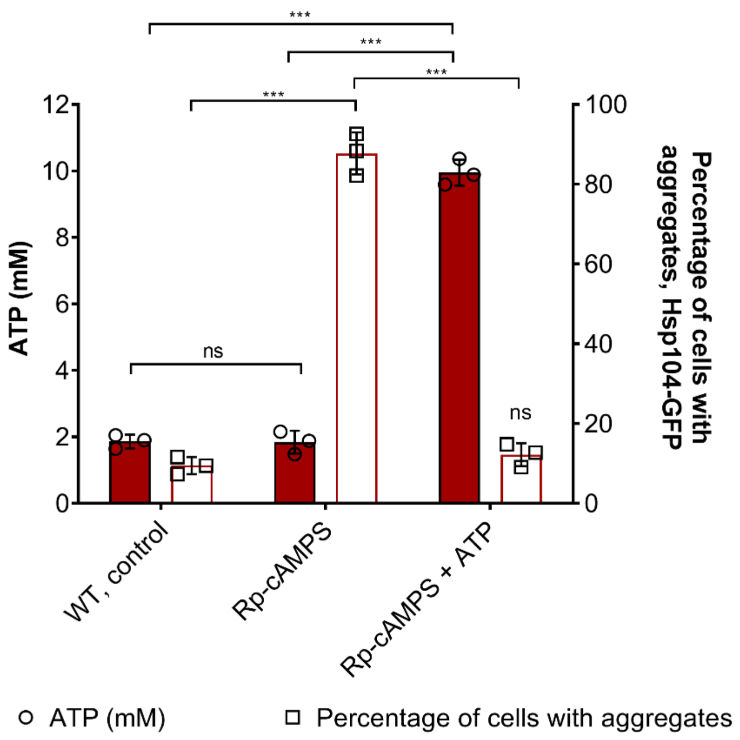

Supplementary Figure 12. The exposure to ATP enables aggregate dissolution during PKA inhibition with Rp-cAMPS during the recovery from glucose starvation. After a 90-minute starvation, cells were placed into 2% glucose, and simultaneously exposed to Rp-cAMPS. 60 minutes later, ATP levels remained low, and aggregates highly abundant. When cells were exposed simultaneously also to ATP (in the presence of DMSO), the intracellular ATP levels increased, while the aggregate abundance decreased to ~10%. ATP levels are represented by red bars and circles, while the percentage of cells with aggregates is represented by white bars and squares. Bar height represents mean ± SD from 3 separate cultures, each performed in triplicate. The mean of three technical replicates for each biological replicate is displayed as single data points. *** $p<0.001$; ** $p<0.01$; * $p<0.05$ (One-way ANOVA plus Tukey post hoc).

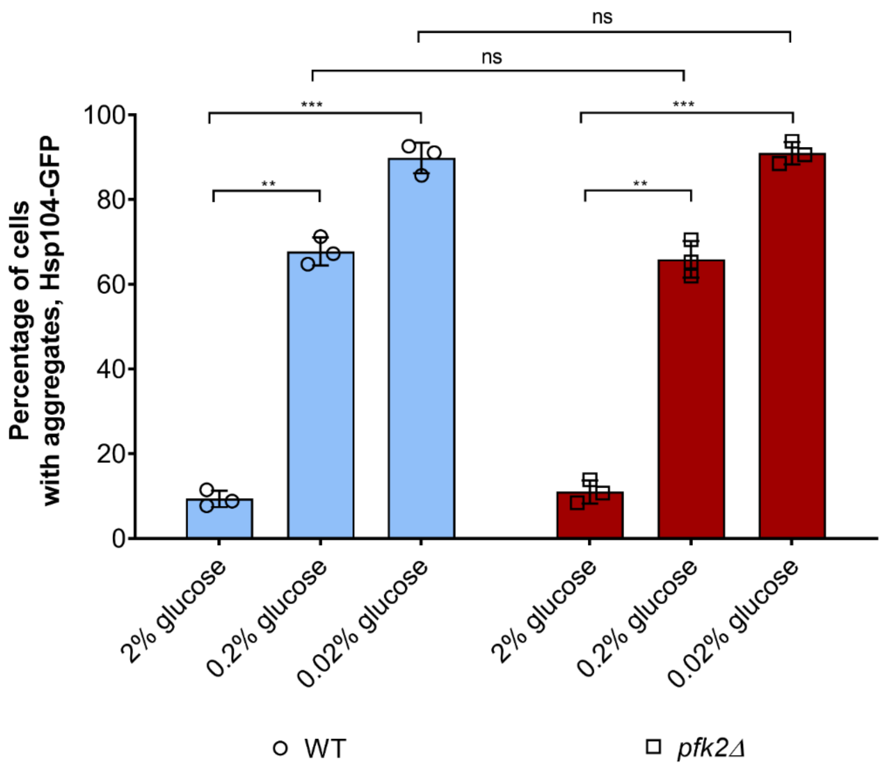

Supplementary Figure 13. Absence of Pfk2 does not affect aggregate abundance during starvation. Aggregate abundance in the WT strain (blue bars, circles), and *pfk2Δ* strain (red bars, squares) is not significantly different. N=550 cells were screened for aggregates starting from three independent exponential yeast cultures for each condition. Data are mean ± SD from at least 3 independent cultures, each performed in triplicate. N=500 cells we screened for aggregates starting from three independent exponential yeast cultures for each condition. Individual data points are mean of three technical replicates for each biological replicate. *** $p<0.001$; ** $p<0.01$; * $p<0.05$ (One-way ANOVA plus post hoc).

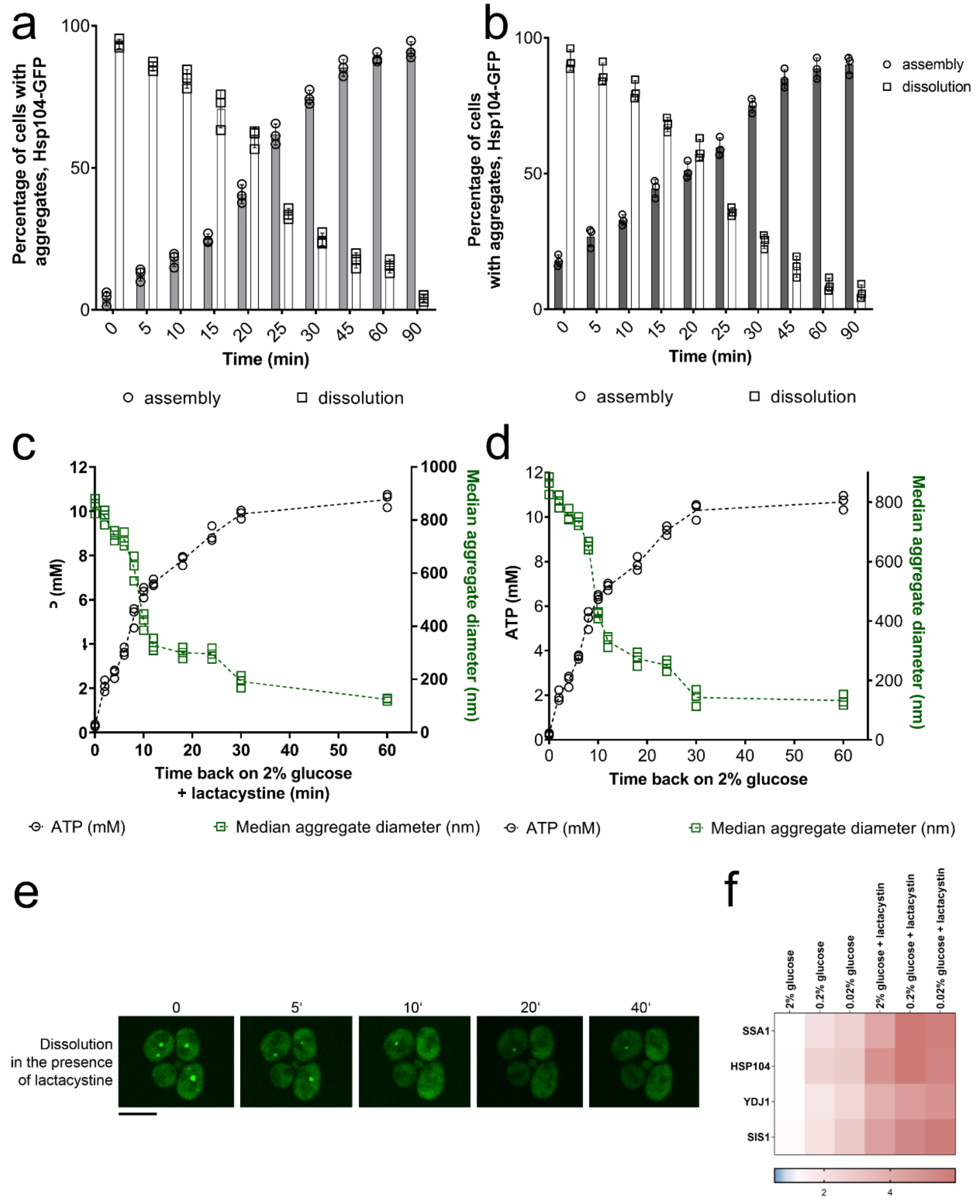

Supplementary Figure 14. Proteasomal inhibition does not affect dissolution of protein aggregates. (a) Percentage of cells displaying at least one Hsp104-GFP tagged aggregate increases during 0.02% glucose starvation, and decreases during recovery in 2% glucose in the presence of lactacystine. Lactacystine was either added at the beginning of starvation, or at the beginning of the recovery period simultaneously with glucose. N=550 cells were screened for aggregates starting from three independent exponential yeast cultures for each condition. Data are mean ± SD from at least 3 independent cultures, each performed in triplicate. Individual data points are mean of three technical replicates for each biological

replicate. **(b)** Cellular ATP (black circles) are restored in 2% glucose, accompanied by a decrease in aggregate diameters (green squares) in the presence of lactacystine. Individual data points are displayed, representing the mean of three technical replicates for three biological replicates. *** p<0.001; ** p<0.01; * p<0.05 (One-way ANOVA plus Tukey post hoc). **(c)** Representative images of aggregate dissolution during the recovery period in the presence of lactacystine. Times at which the images were taken are labeled above them. The images are representative of three technical replicates for each of the three biological replicates (~500 analyzed cells). Black bar represents 8 µm. **(d)** qPCR measurement of differential gene expression of Hsp104, Ssa1, Ydj1, and Sis1. In 2% glucose and during starvation, the presence of lactacytin triggered an increase in expression levels of the tested genes, suggesting the inhibition of proteasomal activity in the applied conditions. Color of the squares on the heat map corresponds to the mean value of the log fold change from three biological and three technical replicates. Standard deviation and p values for the comparison to the control are available in the Source Data file. UBC6 was used for normalization. **(e)** Percentage of cells displaying at least one Hsp104-GFP tagged aggregate increases during 0.02% glucose starvation, and decreases during recovery in 2% glucose in the absence of lactacystine. N=550 cells were screened for aggregates starting from three independent exponential yeast cultures for each condition. Data are mean ± SD from at least 3 independent cultures, each performed in triplicate. Individual data points are mean of three technical replicates for each biological replicate. **(f)** Cellular ATP (black circles) are restored in 2% glucose, accompanied by a decrease in aggregate diameters (green squares) also in the absence of lactacystine. Individual data points are displayed, representing the mean of three technical replicates for three biological replicates. *** p<0.001; ** p<0.01; * p<0.05 (One-way ANOVA plus Tukey post hoc).

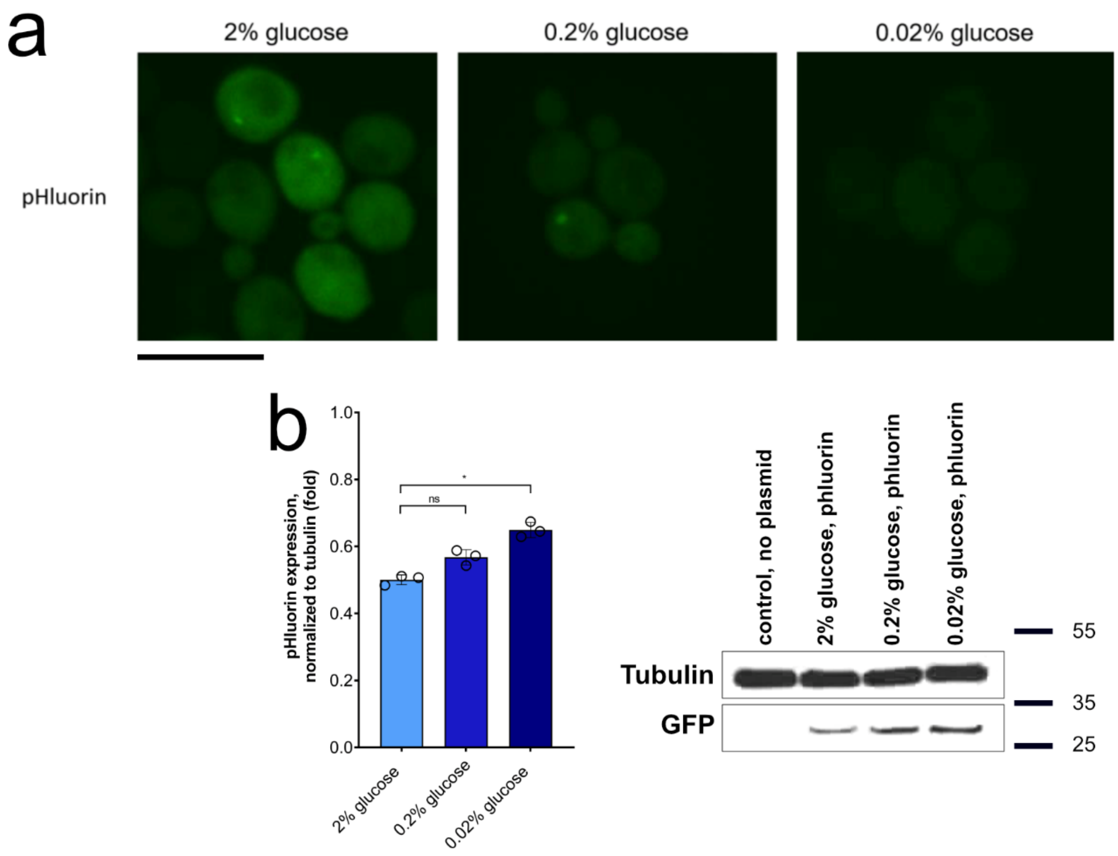

Supplementary Figure 15. Cytosolic pH acidifies during starvation. (a) pH of the cytosol is decreasing with the decline in the glucose concentration, observed as a decrease in the fluorescence intensity of the pH sensitive variant of GFP (pHluorin). The images are representative of three technical replicates for each of the three biological replicates (~500 analyzed cells). Black bar represents 8 µm. **(b)** Western blot measurement of the expression level of the pH sensitive variant of GFP shows a slight increase of GFP protein level at 0.02% glucose, compared to 2% glucose. Cells were exposed to starvation in mid-exponential phase. The control cells underwent the same centrifugation treatment as the cells that were exposed to starvation. Bar height represents mean ± SD from 3 separate cultures, each performed in triplicate. Representative blot of three independent biological replicates is shown. Individual data points are displayed, representing the result of three biological replicates. *** $p<0.001$; ** $p<0.01$; * $p<0.05$ (One-way ANOVA plus Tukey post hoc).

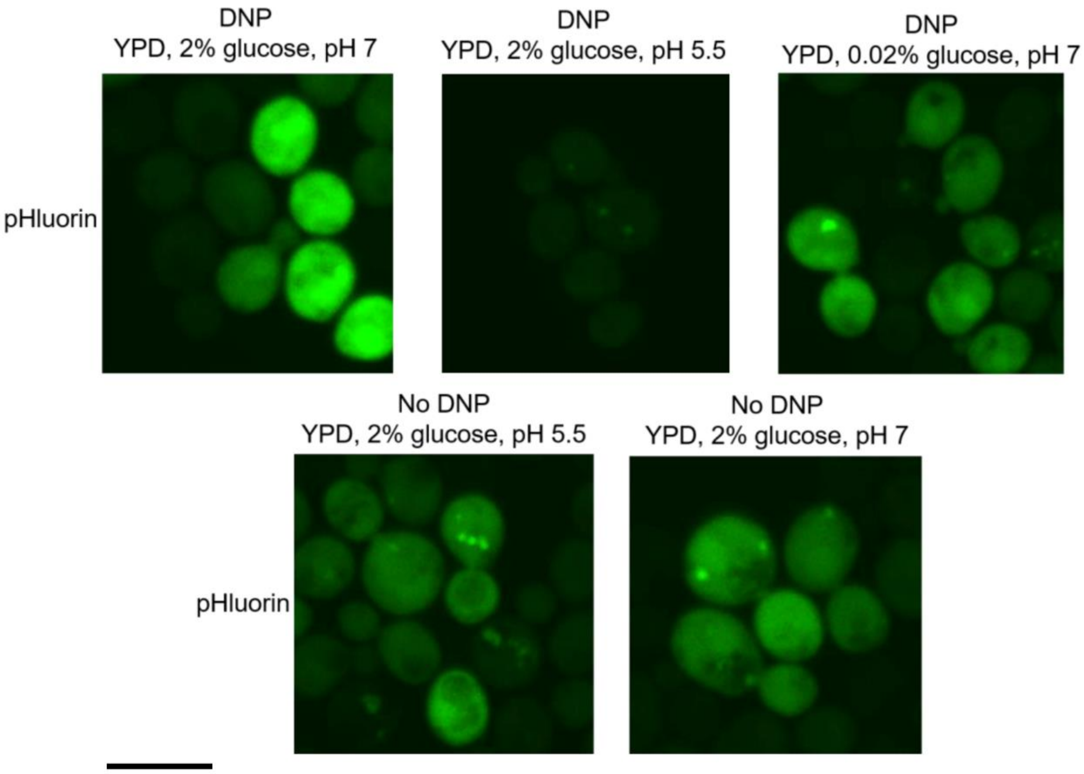

Supplementary Figure 16. DNP enables the equilibration of pH between medium and cytosol. In the presence of DNP the pH of the cytosol adopts the pH of the medium (top row), as observed using the pH sensitive variant of GFP (pHluorin). In the absence of DNP in 2% glucose the cytosol remains neutral, regardless of the pH of the medium (bottom row). The images are representative of three technical replicates for each of the three biological replicates. Black bar represents 8 µm.

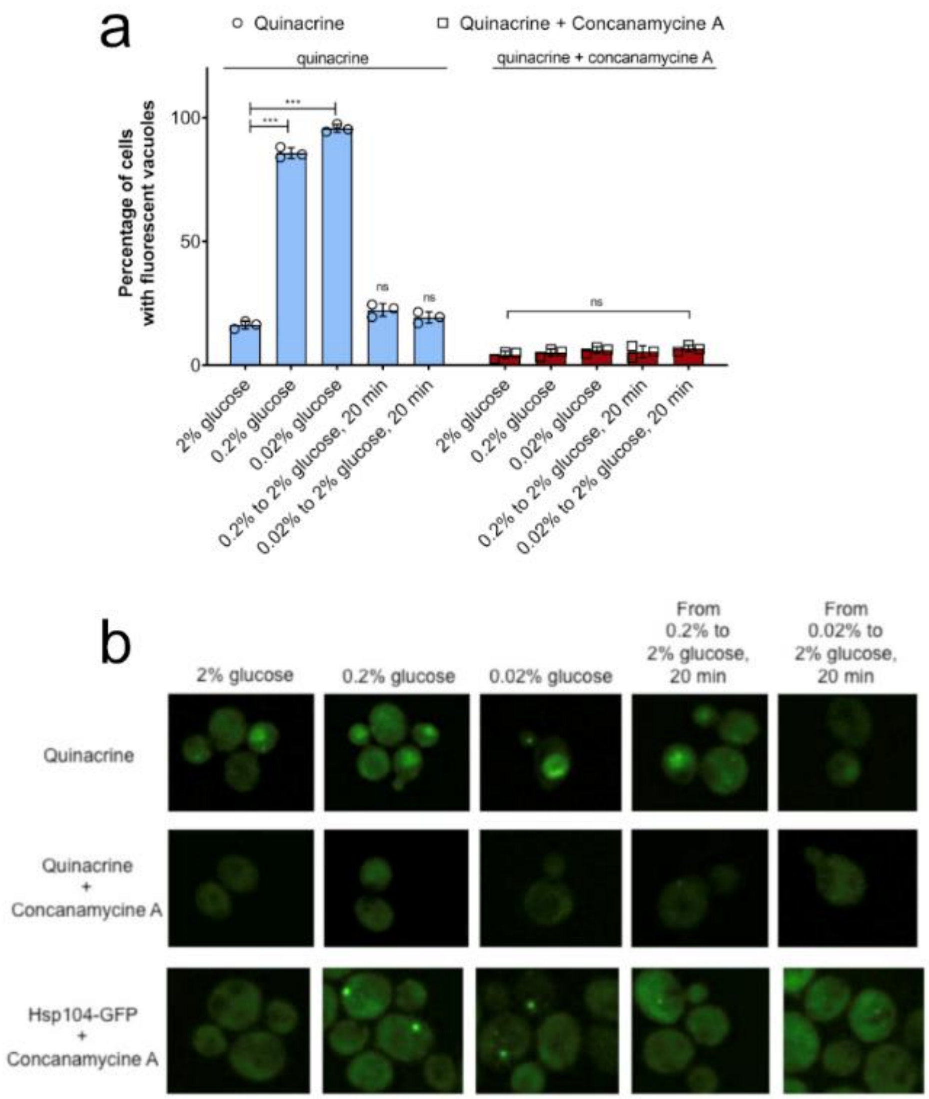

Supplementary Figure 17. Quinacrine inhibits vacuolar acidification during starvation. (a) Percentage of cells with acidic vacuolar pH increases during starvation and is inhibited by concanamycine A. Increase in quinacrine fluorescence in the vacuole suggests a decrease in vacuolar pH during starvation. The starvation was performed in mid-exponential phase for 90 minutes. Prior to the onset of starvation, the cells were exposed to quinacrine and concanamycine A. N=1200 cells were screened for vacuolar staining starting from three independent exponential yeast cultures for each condition. Data are mean ± SD from at least 3 independent cultures, each performed in triplicate. *** $p<0.001$; ** $p<0.01$; * $p<0.05$ (One-way ANOVA plus Tukey post hoc). Black bar represents 8 µm. **(b)** Representative images. Inhibition of vacuolar acidification by concanamycine A did not affect aggregate formation or dissolution. The images are representative of three technical replicates for each of three biological replicate.

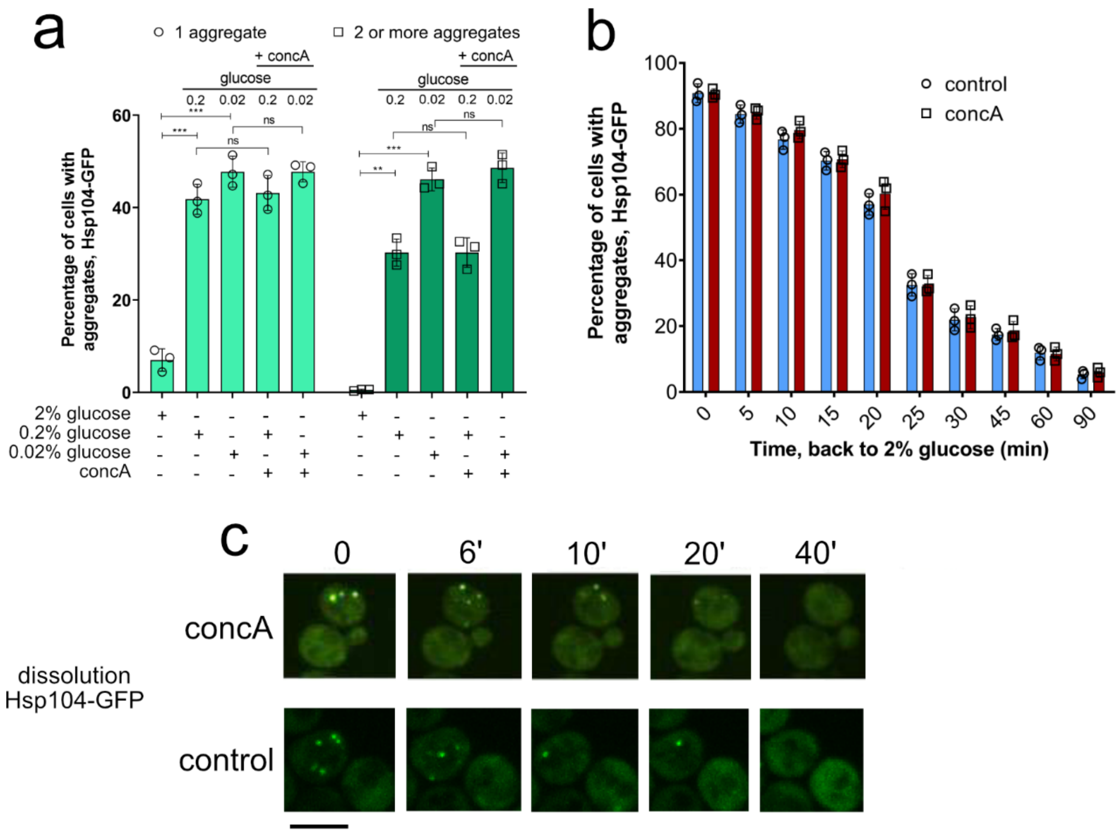

Supplementary Figure 18. Vacuolar acidification does not contribute to the aggregate dissolution after glucose starvation. (a) In 0.2% glucose, around 40% of cells have one aggregate, while around 30% have two or more. In 0.02% glucose close to 50% of cells have one, and a comparable number has two or more aggregates. These values are not significantly changed due to concanamycine A (concA) treatment. N=1200 cells were screened for aggregates starting from three independent exponential yeast cultures for each condition. **(b)** Concanamycine A did not affect protein dissolution kinetics during post starvation recovery. N=1200 cells were screened for aggregates starting from three independent exponential yeast cultures for each condition. Bar height represents mean ± SD from 3 separate cultures, each performed in triplicate. The mean of three technical replicates for each biological replicate is displayed as single data points. *** $p<0.001$; ** $p<0.01$; * $p<0.05$ (One-way ANOVA plus Tukey post hoc). **(c)** Representative images are displayed for the aggregate dissolution in the absence and presence of concA. The images are representative of three technical replicates for each of the three biological replicates. Black bar represents 8 µm.

Supplementary Table 1. The list of strains used in this study, their genotype and source.

Strain	Genotype	Source	Growth medium
	MATa his3Δ1 leu2Δ0 met15Δ0 ura3Δ0 Hsp104-GFP:His3Mx6	Thermo Fisher Scientific	YPD
	MATa his3Δ1 leu2Δ0 met15Δ0 ura3Δ0 Hsp42-GFP:His3Mx6	Thermo Fisher Scientific	YPD
yYB5658	MATalpha ade2::hisG his3 leu2 met15D::ADE2 ura3D0 trp1D63 hoD::SCW11pr-Cre-EBD78-NatMX loxP-UBC9-loxP-LEU2 loxP-CDC20-Intron-loxP-HPHMX HSP104-Cherry:KanMX	Courtesy of Prof. Yves Barral	YPD
yYB5841	MATa ade2::hisG his3 leu2 met15D::ADE2 ura3D0 trp1D63 hoD::SCW11pr-Cre-EBD78-NatMX loxP-UBC9-loxP-LEU2 loxP-CDC20-Intron-loxP-HPHMX SSA1-GFP:HIS3 HSP104-Cherry:KanMX	Courtesy of Prof. Yves Barral	YPD
yYB11406	MATa ade2::hisG his3 leu2 met15D::ADE2 ura3D0 trp1D63 hoD::SCW11pr-Cre-EBD78-NatMX loxP-UBC9-loxP-LEU2 loxP-CDC20-Intron-loxP-HPHMX Hsp82-GFP:HIS3 HSP104-Cherry:KanMX	Courtesy of Prof. Yves Barral	YPD
yYB11531	MATa/MATα ADE2/ade2-101 ADE1/ade1-4 his3/ his3-11 leu2/leu2-3 ura3D0/ura3-1 trp1D63/trp1-1 hoD::SCW11pr-Cre-EBD78-NatMX loxP-UBC9-loxP-LEU2 loxP-CDC20-Intron-loxP-HPHMX Sup35-GFP:HIS3/+ HSP104-mCherry:KanMX/+ [PIN+] [psi-]	Courtesy of Prof. Yves Barral	YPD
AK01	MATα SUC2 gal2Δ mal2Δ melΔ flo1Δ flo8-1 hap1Δ hoΔ bio1Δ bio6Δ + pPGK-pHluorin::URA3	This work	Ura DO
US01	MATa his3Δ1 leu2Δ0 met15Δ0 ura3Δ0 HSP104-GFP:His3Mx6 hsp42Δ::Hyg	This work	YPD
US02	MATa his3Δ1 leu2Δ0 met15Δ0 ura3Δ0 HSP104-GFP:His3Mx6 hsp104Δ::Hyg	This work	YPD
US03	MATa his3Δ1 leu2Δ0 met15Δ0 ura3Δ0 hsp104Δ::Hyg::TEF1-HSP104^{E285Q}-GFP::URA3	This work	YPD
US04	MATa his3Δ1 leu2Δ0 met15Δ0 ura3Δ0 hsp104Δ::Hyg::TEF1-HSP104^{E687Q}-GFP::URA3	This work	YPD
US05	MATa his3Δ1 leu2Δ0 met15Δ0 ura3Δ0 hsp104Δ::Hyg::TEF1-HSP104-GFP::URA3	This work	YPD
US06	MATa his3Δ1 leu2Δ0 met15Δ0 ura3Δ0 HSP104-GFP:His3Mx6 + pTDH3myc-RNQ1-mCherry::URA3	This work	Ura DO
US07	MATa his3Δ1 leu2Δ0 met15Δ0 ura3Δ0 HSP104-GFP:His3Mx6 + pTDH3myc-NRP1-mCherry::URA3	This work	Ura DO

Supplementary Table 2. Primers used in this study.

Amplification of sequences upstream and downstream of *HSP104*

Hsp104-u-fwd	ACA TGC GGT TGT GGC GAG AG
Hsp104-u-rc	AT CAGCTG ATA TTC TGT ATA TTT TAT GGT ACG TGT AG
Hsp104-d-fwd	AT GAGCTC TTT AAT ATA GTG TGA TTT TTA AAA ACT TTC GAA
Hsp104-d-rc	ATT ACA TGA ACT GCT ATG GTA AAA ATT TAA

Hygromycine cassette amplification

HygB-fwd	CGTACGCTGCAGGTCGAC
HygB-rc	ATCGATGAATTCGAGCTCG

Amplification of sequences upstream and downstream of *HSP42*

Hsp42-u-fwd	AATATTTAGCTGGGGTTGGGTAAC
Hsp42-u-rc	TATCAGCTGTGCTTCGGCTTGGTATGAT
Hsp42-d-fwd	TATGAGCTCATATCGTATCTGTTTATACAC
Hsp42-d-rc	ACAGTACAATGGAGTCTTTCAAGA

Rnq1 amplification

Rnq1-fwd	GA GGATCC ATG GAT ACG GAT AAG TTA ATC TCA GA
Rnq1-rc	GA CTC GAG TCA GTA GCG GTT CTG GTT GC

Nrp1 amplification

Nrp1_fwd	GA GGATCC ATG CAC TAT GTG GTA CTA GAG CT
Nrp1_rc	GA CTC GAG CTA CCA ACG TAT TGA ACT ATT AAA AC

Primers used for genomic integrations of Hsp104 WT and the mutant forms

TDH3-SpeI-FP	5’- GGACTAGTGGCCTATGCGGCCCAGTTCG-3’
TDH3-EcoRI-RP	5’-GGAATTCGGATCCAAGCTTTTTGTTTATGTGTGTTTATTCGAAACTA-3’
EGFP-EcoRI-FP	5’- GGAATTCGGTACCGTCGACACCGGTATGGTGAGCAAGGGCGAGGAGCT [illegible]
EGFP-XhoI-RP	5’-CCGCTCGAGTTACTTGTACAGCTCGTC-3’
HSP104-EcoRI-FP	5’-CGCGGATCCAATGAACGACCAAACGCAATT-3’
HSP104-XhoI-RP	5’-CCGCTCGAGATCTAGGTCATCATCAATTTCC-3’
HIS3-FP	5’-GCGGGATTGCTCTCG-3’
HIS3-RP	5’-AGTCTTCAGTGGTGTGATG-3’
